# Opposite Expression of Hepatic and Pulmonary Corticosteroid-Binding Globulin in Cystic Fibrosis Patients

**DOI:** 10.3389/fphar.2018.00545

**Published:** 2018-06-05

**Authors:** Anastasia Tchoukaev, Jessica Taytard, Nathalie Rousselet, Carine Rebeyrol, Dominique Debray, Sabine Blouquit-Laye, Marie-Pierre Moisan, Aline Foury, Loic Guillot, Harriet Corvol, Olivier Tabary, Philippe Le Rouzic

**Affiliations:** ^1^INSERM, Centre de Recherche Saint-Antoine, Sorbonne Université, Paris, France; ^2^Pediatric Respiratory Department, Trousseau Hospital, Assistance Publique – Hôpitaux de Paris, Paris, France; ^3^Pediatric Hepatology Unit, Necker Enfants Malades Hospital, Paris, France; ^4^INSERM U1173, UFR des Sciences de la Santé Simone Veil, Université de Versailles Saint-Quentin-en-Yvelines, Versailles, France; ^5^INRA, Laboratoire NutriNeurO, UMR 1286, Université de Bordeaux, Bordeaux, France

**Keywords:** corticosteroid-binding globulin, cystic fibrosis, glucocorticoids, liver, lung

## Abstract

Cystic fibrosis (CF) is characterized by a chronic pulmonary inflammation. In CF, glucocorticoids (GC) are widely used, but their efficacy and benefit/risk ratio are still debated. In plasma, corticosteroid-binding globulin (CBG) binds 90% of GC and delivers them to the inflammatory site. The main goal of this work was to study CBG expression in CF patients in order to determine whether CBG could be used to optimize GC treatment. The expression of CBG was measured in liver samples from CF cirrhotic and non-CF cirrhotic patients by qPCR and Western blot and in lung samples from non-CF and CF patients by qPCR. CBG binding assays with ^3^H-cortisol and the measurement of the elastase/α1-antitrypsin complex were performed using the plasmas. CBG expression increased in the liver at the transcript and protein level but not in the plasma of CF patients. This is possibly due to an increase of plasmatic elastase. We demonstrated that pulmonary CBG was expressed in the bronchi and bronchioles and its expression decreased in the CF lungs, at both levels studied. Despite the opposite expression of hepatic and pulmonary CBG in CF patients, the concentration of CBG in the plasma was normal. Thus, CBG might be useful to deliver an optimized synthetic GC displaying high affinity for CBG to the main inflammatory site in the context of CF, e.g., the lung.

## Introduction

Cystic fibrosis (CF) is the most frequent autosomal recessive genetic disorder among the Caucasian population. This disease is caused by mutations in the *CFTR* gene (Cystic Fibrosis Transmembrane conductance Regulator) which encodes a chloride channel (Riordan et al., 1989). Due to the wide expression of CFTR, CF is a multi-organ disease, affecting the lungs, the liver, the digestive tract, the pancreas, the reproductive tract and the sweat glands.

In the liver, CFTR is expressed at the apical membrane of the cholangiocytes (Cohn et al., 1993). Its absence or dysfunction leads to a decrease in bile flow, and the retention of endogenous hydrophobic bile acids that may be responsible for cell membrane injury (Debray et al., 2017). This, in turn, causes inflammation and collagen deposition around the bile ducts and portal tracts, leading to focal biliary and periportal fibrosis, which can progress to multilobular cirrhosis with portal hypertension (Feranchak and Sokol, 2001). The German registry reports that up to 32% of CF patients develop liver disease (Nährlich et al., 2016). Lung disease still remains the main cause of morbidity and mortality in CF patients. In CF airways, the lack of functional CFTR indeed leads to the stagnation of an abnormally thick mucus, which is favorable to pathogens colonization. Characteristic inflammation and infection cycles appear, inducing a progressive pulmonary degradation, and, eventually, lead to lung failure (Jacquot et al., 2008; Pittman and Ferkol, 2015). Chronic and poorly controlled inflammation, reinforced by an important bacterial colonization, contributes to the deterioration of the epithelium in CF airways.

Anti-inflammatory treatments, primarily glucocorticoids (GC), represent one of the main tools for the practitioner to delay lung injury. In France, 37.3% of CF patients were treated with inhaled GC in 2015 and more than 16% with oral GC (Vaincre la Mucoviscidose and Ined, 2017). Nevertheless, the use of GC is still controversial in the CF context. Indeed, a multi-centric study failed to show any beneficial effects, whatsoever, of inhaled GC in CF patients and highlighted the fact that at high doses, they can cause adverse effects (Balfour-Lynn and Welch, 2016). In 1985, a pioneer study showed that oral GC could help in preventing the decrease of the pulmonary function in CF patients (Auerbach et al., 1985), but these results were quickly contradicted by other studies showing major side effects associated with a long-term use (Eigen et al., 1995; Lai et al., 1999). More recently, a multi-centric study on the use of oral GC confirmed those side effects (Cheng et al., 2015). Controversially, previous works from our laboratory showed that, *in vitro*, GC are efficient to reduce the inflammation in bronchial epithelial CF cells (Rebeyrol et al., 2012). A better understanding of the mode of action of GC is therefore needed in the context of CF. We were interested in particular on their extracellular regulation through their specific binding globulin, corticosteroid-binding globulin (CBG), which is poorly studied.

Corticosteroid-binding globulin, or SERPINA6, is a glycoprotein that is mainly produced by the liver, and more specifically by the hepatocyte (Khan et al., 1984). It is released in the plasma where it binds approximately 90% of GC. At the inflammatory site, CBG is cleaved by activated neutrophils elastase, leading to a conformational change of CBG and resulting in the local release of bound GC (Pemberton et al., 1988; Hammond, 1990). Recent studies show that CBG is also cleaved specifically by elastase (LasB) from *Pseudomonas aeruginosa*, which represents the main bacteria found in CF airways (Simard et al., 2014). CBG is therefore the protein that addresses the GC to the inflammatory and infection site and, in particular, to the lung in the context of CF. Furthermore, some studies show that CBG is also produced by other organs, including the lung (Hammond et al., 1987). This local expression could modulate the efficiency of GC in the lung, the main site of inflammation in CF patients, but its function and pattern of expression are still unknown.

The goal of this study was to investigate the expression of hepatic and pulmonary CBG in CF patients in order to determine if CBG could be used to optimize GC treatment.

## Materials and Methods

### Subjects and Samples

Liver and lung specimens as well as blood samples were collected and processed in compliance with the standard guidelines for human research (Declaration of Helsinki) and the current French public health legislation (articles L.1235-2 and L.1245-2, code de la santé publique^1^). Written consent for using the liver or lung tissue samples for research purposes was obtained from the patients or parents.

The liver samples were obtained during liver transplantation from 9 CF cirrhotic (cir) patients (18 ± 2 years old, seven men and two women) and 5 non-CF cir patients (22 ± 2 years old, three men and two women), who developed cirrhosis due to biliary atresia (BA).

The lung lobes were obtained from 10 non-CF patients who underwent surgery (61 ± 6 years old, 10 men) and from 19 CF patients (30 ± 7 years old, 10 men and 9 women) who underwent lung transplantation. The samples from the non-CF patients were obtained from a non-pathologic area without any inflammation. After dissection of the tissue, the samples were directly frozen in liquid nitrogen before extraction.

The blood samples were collected after obtaining an informed consent from each patient included in the study, including: 32 non-CF patients (22 ± 7 years old, 13 men and 19 women) with 22 young healthy adults and 10 non-CF cir patients (8 BA and 2 autoimmune cirrhosis); and 31 CF patients (13 ± 3 years old, 21 men and 10 women) with 10 cir patients, 8 patients without liver involvement, 12 with focal fibrosis and 1 with liver steatosis. The blood samples were immediately centrifuged for 15 min at 4,000 rpm at 4°C. The plasma was aliquoted and kept at -80°C until use.

### RNA Extraction

The hepatic (500 mg) or lung samples (100 mg) were grinded using the Polytron device (PT 3,100 Kinematica, Luzern, Switzerland) at high speed in 1 mL of TRIzol^TM^ (Thermo Fisher Scientific, Illkirch-Graffenstaden, France). After that, we followed the modified Chomczynski and Sacchi protocol (Chomczynski and Sacchi, 1987). The RNA pellets were finally dissolved in 50 to 100 μL of sterile water.

### Reverse Transcription (RT) and Quantitative PCR (qPCR)

The total RNA (1 μg) was heated at 65°C for 5 min and was then cooled down on ice. After that, we followed Promega’s recommendations for the RT (Charbonnières-les-Bains, France). Then, the mix was incubated for 1 h at 37°C.

The transcripts levels were assessed by qPCR using CBG (Hs00156318_m1), GAPDH (Hs02786624_g1), and 18S (Hs03928992_g1) TaqMan^TM^ probes obtained from Applied Biosystems (Thermo Fisher Scientific). The *CBG* transcripts were normalized to the *18S* transcripts levels in the hepatic samples and to the *GAPDH* transcripts levels in the pulmonary samples. The experiments were performed in duplicate for each qPCR point. A total of 1 μL of RT product was mixed with TaqMan^TM^ Fast Universal PCR Master Mix 2X (Thermo Fisher Scientific) for a final volume of 10 μL. The qPCR experiments were done using Thermo fisher’s thermocycler StepOnePlus and were analyzed with StepOne Software v2.2.2.

### Protein Extraction and Western Blot

Cubes of livers (3 mm) were grinded in lysis buffer (Tris–HCl pH 7.5 10 mM, NaCl 150 mM, EDTA 3 mM, NP-40 1%, deoxycholate 0.5%, and SDS 0.1%). The samples were agitated at 1,300 rpm at 4°C for 30 min and then centrifuged at 15,000 *g* for 10 min at 4°C. The upper phase was collected, and the protein concentrations were assessed by a BCA protein assay kit (Thermo Fisher Scientific).

The livers proteins (30 μg) were reduced in lysis buffer with Laemmli 1× and β-mercaptoethanol (1/5) by heating for 5 min at 100°C and were separated on 10% SDS-polyacrylamide gels. Then, the proteins were transferred to nitrocellulose membranes (iBlot^TM^ transfer system, Thermo Fisher Scientific). Non-specific sites were blocked for 1 h in 5% milk in Tris buffer saline tween (TBS-T). The membranes were incubated overnight at 4°C with a rabbit polyclonal anti-human CBG antibody (1/2,500), kindly provided by Dr. John G. Lewis (Christchurch, New Zealand) or a mouse anti-human β-actin antibody (A2228, Sigma-Aldrich, Saint-Quentin-Fallavier, France; 1/20,000) for 1 h. The membranes were then incubated for 1 h with an anti-rabbit IgG HRP-linked antibody (1/5,000) from Cell Signaling (Leiden, Netherlands; reference: 7074) or an anti-mouse IgG HRP-linked antibody (1/5,000; reference: 7076). The chemiluminescent images were captured using the Chemidoc Touch (Bio-Rad, Marnes-la-Coquette, France) following exposure to the SuperSignal West Femto kit (Thermo Fisher Scientific). The quantifications were made using ImageJ software.

### CBG Binding Assay

The binding capacity of CBG for cortisol was measured at 4°C by a solid phase binding assay using concanavalin A-sepharose (modified from Pugeat et al., 1984) (reference: 17-0440-01, Dutscher, Brumath, France). Briefly, the endogenous glucocorticoid from plasma free of fibrin was first absorbed onto activated charcoal (plasma/charcoal vol/vol) by agitation for 30 min at room temperature followed by a centrifugation at 10,000 rpm at 4°C for 15 min. CBG was then adsorbed onto a solid phase matrix of con A-sepharose by incubating it for 30 min in 50 μL of stripped plasma with 250 μL of conA-sepharose. Each tube was then washed, and after centrifugation for 15 min at 4,000 rpm at 4°C, the aqueous phase containing the non-adsorbed proteins, such as albumin, was removed. ^3^H-cortisol (30,000 cpm, Perkin Elmer, Villebon-sur-Yvette, France, ref: NET-396) and increasing concentrations of unlabelled cortisol (0, 1.25, 2.5, 5, 10, 20, and 500 ng/tube, reference: H-4001, Sigma-Aldrich) were added. The mixture was agitated and incubated for 45 min at 4°C with mixing before being centrifuged at 4,000 rpm for 15 min at 4°C. The upper phase was removed and 2.5 mL of scintillate liquid was added [Ecolite(+)^TM^, MP Biomedicals, Illkirch-Graffenstaden, France]. Every tube was counted for 3 min by a Hidex 300 SL detector (Hidex, Turku, Finland).

The binding capacity of CBG for cortisol was calculated by a Scatchard analysis using “bound” as the quantity of cortisol specifically bound to the glycoproteins adsorbed to the gel and “free” as the concentration of cortisol in the aqueous phase.

### Elastase/α1-Antitrypsin Complex Measurements

The concentration of the elastase/α1-antitrypsin (AAT) complex in the plasmas was measured according to the manufacturer’s recommendations (QIA96, Sigma-Aldrich).

### Statistical Analysis

The horizontal bar on each graph represents the mean. The outliers were identified by Grubbs’ test^2^. The data were compared using a Student’s *t*-test for comparing two groups and a one-way ANOVA followed by Bonferroni’s post-test for comparing more than two groups. All the analyses were performed on GraphPad Prism version 7 software: *p* < 0.05 was considered significant. For each graph, the significant difference is represented as: ^∗^*p* < 0.05, ^∗∗^*p* < 0.01, and ^∗∗∗^*p* < 0.001.

## Results

### Expression of CBG in the Livers of CF Patients

**FIGURE 1:**
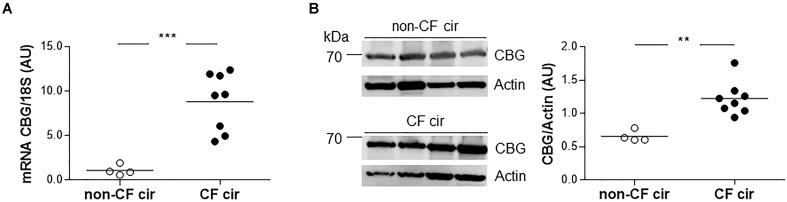
Corticosteroid-binding globulin (CBG) expression in the liver of CF and non-CF cirrhotic patients. **(A)** The transcriptional expression of *CBG* was normalized to *18S* in the non-CF cirrhotic (non-CF cir, *n* = 4) and CF cirrhotic (CF cir, *n* = 8) livers. Each point represents the relative quantity of *CBG* transcripts normalized to the mean of the non-CF cir patients. **(B)** CBG protein expression was measured by a Western blot and was normalized to β-actin expression in the non-CF cirrhotic (non-CF cir, *n* = 4) and CF cirrhotic (CF cir, *n* = 8) livers. Each point represents the quantity of CBG normalized to β-actin for each patient. The horizontal bar represents the mean for each group. The data were compared using an unpaired Student’s *t*-test (^∗∗^*p* < 0.01 and ^∗∗∗^*p* < 0.001).

Since the main CBG production site is in the liver, we first studied the expression of hepatic CBG in liver tissue from CF cirrhotic (cir) and non-CF cir patients. A significant eightfold increase in CBG transcripts was found in the CF cir livers compared to the non-CF cir livers with a significant *p*-value < 0.0001 (**Figure 1A**). The variation between these groups was confirmed at the protein level by a Western blot (80% of increase; *p* < 0.01) (**Figure 1B**).

### CBG and Elastase/α1-Antitrypsin Complex Measurements in the Plasma of CF Patients

**FIGURE 2:**
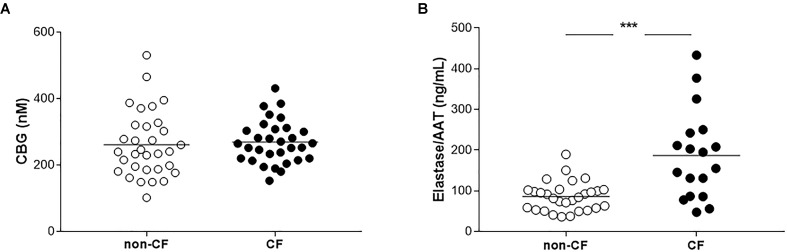
Measurements of CBG and elastase/AAT complex in plasma of non-CF and CF patients. **(A)** CBG concentrations measurements by a binding assay with ^3^H-cortisol in the plasmas of non-CF patients (healthy or cirrhotic subjects, *n* = 32) and CF patients (with a liver involvement or not, *n* = 31). Each point represents the concentration of CBG for each patient. **(B)** Elastase/AAT complex measurements in the plasmas of non-CF patients (healthy or cirrhotic subjects, *n* = 29) and CF patients (with a liver involvement or not, *n* = 18). Each point represents the concentration of the elastase/AAT complex for each patient. The mean for each group is represented by a horizontal bar. The data were analyzed by a one-way ANOVA followed by Bonferroni’s *post hoc* test (^∗∗∗^*p* < 0.001).

To assess if the increase in CBG in the liver of CF patients resulted in an increase in the protein release into the plasma, we measured the concentration of CBG in the plasma of CF and non-CF patients using a binding assay with concanavalin A-sepharose. For all the subjects, the mean concentration of CBG was similar without any significant differences (non-CF: 260.2 ± 98.1 nM; CF: 270.3 ± 64.4 nM) (**Figure 2A**).

Because neutrophils elastase cleaves CBG and is thus responsible for the release of GC, we therefore measured the plasmatic concentration of elastase in the two groups. We found a significant twofold increase of the mean of elastase/AAT complex in the plasma of the CF patients compared to the non-CF, 186.83 ng/mL vs. 85.92 ng/mL (*p* < 0.0001) (**Figure 2B**).

### Pattern of Expression of Pulmonary CBG

**FIGURE 3:**
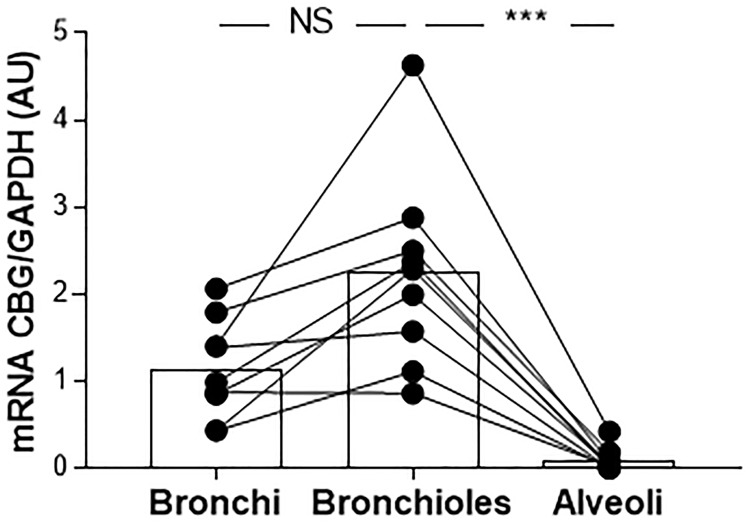
The pattern of expression of pulmonary CBG. *CBG* mRNA expression was analyzed by RT-PCR and was normalized to *GAPDH* mRNA expression in the bronchial, bronchiolar, and alveolar biopsies from 9 non-CF patients (healthy parts of lung from patients with non-CF pulmonary pathologies). Each point represents the relative quantity of the *CBG* transcripts normalized to the bronchial mean. The linked points represent the data collected for the same patient. The histogram represents the mean for each group. The data were analyzed by a one-way ANOVA followed by Bonferroni’s *post hoc* test (^∗∗∗^*p* < 0.001). NS, not significant.

Because the main site of inflammation in CF is the lung, we evaluated the expression of CBG in the lung tissue. We first studied the pattern of CBG expression in the lung of the non-CF patients. Due to the possible contamination by the hepatic CBG protein that could circulate and be found in the lung, especially considering the low pulmonary expression compared to the hepatic one, we only measured the local expression of CBG by real-time quantitative PCR. *CBG* mRNA expression was measured in the bronchial, bronchiolar, and alveolar biopsies. We found the expression of CBG in the bronchi and bronchioles (mean expression in arbitrary unit: 1.14 and 2.25, respectively) while barely any *CBG* mRNA was detectable in the alveoli (mean expression in arbitrary unit: 0.09 with a *p* < 0.0001) (**Figure 3**).

### Expression of CBG in the Lungs of CF Patients

**FIGURE 4:**
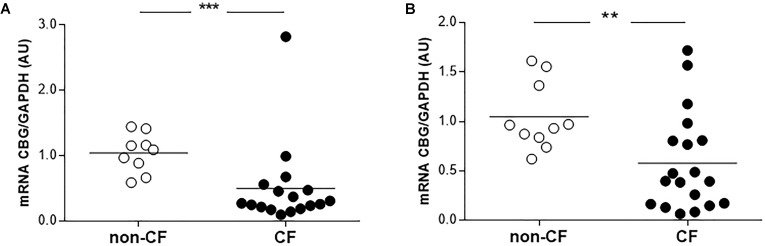
Expression of pulmonary CBG in the lungs of CF patients. **(A)** The transcriptional analysis of *CBG* was normalized to *GAPDH* in the bronchi of CF (*n* = 17) and non-CF patients (*n* = 9). **(B)** The transcriptional analysis of *CBG* was normalized to *GAPDH* expressions in the bronchioles of CF (*n* = 19) and non-CF patients (*n* = 10). Each point represents the relative quantity of the *CBG* transcripts normalized to the non-CF mean. The mean for each group is represented by a horizontal bar. The data were compared using an unpaired Student’s *t*-test (^∗∗^*p* < 0.01 and ^∗∗∗^*p* < 0.001).

Finally, we compared the CBG expression between the non-CF and CF patients in the bronchial and bronchiolar biopsies, since CBG was not expressed in the alveolus. The *CBG* transcripts expression was decreased in the bronchi (by 52%, *p* < 0.001) and bronchioles (by 45%, *p* < 0.01) of the CF patients compared to the non-CF patients (**Figure 4**).

## Discussion

Despite serious side effects, GC represent one of the main anti-inflammatory therapies for CF patients. Given that CBG could be used to deliver GC specifically to the inflammatory site (e.g., the lung in CF), the aim of this work was to study the hepatic and pulmonary CBG expression in CF patients. CBG was never studied in this context, even though it could lead to the optimization of GC treatment for CF patients. In this study, we demonstrated that the CBG expression in CF patients was upregulated in the liver and downregulated in the lung, while the plasmatic level remained unchanged compared to the non-CF patients.

We showed an increase in the CBG transcripts and protein in the liver of the CF patients (**Figure 1**). A pioneer study suggested a slight decrease in plasmatic CBG capacity in CF patients who had low liver function, presumably due to disturbed protein synthesis by the liver (Nowaczynski et al., 1987). Our findings do not support this assessment. To our knowledge, no increase in CBG expression in the liver was ever described in a pathological context. Cirrhotic livers from CF and non-CF patients were chosen to compare, at best, the results, and it seems that this increase is therefore specific of the context of CF. However, because CBG is expressed and secreted by hepatocytes (Khan et al., 1984), whereas CFTR is exclusively expressed by another cell type, cholangiocytes (Cohn et al., 1993), this increase does not seem to be the direct consequence of a dysfunction of the CFTR channel within the cell. We hypothesize that it would rather be the specific microenvironment of the CF liver that would be responsible for this regulation. Indeed, the cholangiocyte abnormalities caused by CF, leading to toxic bile acids accumulation in the liver, are responsible for inflammation with the release of proinflammatory cytokines, growth factors and the activation of hepatic stellate cells to synthesize collagen (Leeuwen et al., 2014). The particular environment and architectural reshaping of the liver in CF patients is unique, not resembling any other liver pathologies (Staufer et al., 2014). The pathogenesis of CF liver disease has still largely been unknown. Further investigation about the physiopathology of CF liver disease would be fundamental to better understand the new regulation we observed here.

We then focused our work on the level of plasmatic CBG, which, if it is enough, could be used to deliver GC in CF patients. Some studies show a decreased in CBG concentration in the plasma in a pathological context, such as in burned patients (Bernier et al., 1998), during a septic shock (Pugeat et al., 1989) or in the plasma of patients with metabolic disorders (Fernandez-Real et al., 2001). A decreased in CBG concentrations was also described in the plasma of cirrhotic patients, but these studies included heterogeneous groups of patients with various causes of cirrhosis other than BA (Coolens et al., 1987; McDonald et al., 1993; Wiest et al., 2008). This could explain why we did not see this decrease in our cohort (data not shown). We showed that the mean concentration of CBG in CF patients was the same as the control group (**Figure 2A**). This specific and interesting result, considering the increase of CBG in the liver of CF patients, lead us to two hypotheses, including that the absence of an increase in plasmatic CBG could be explained by hepatic retention and/or the increased cleavage of CBG in the plasma. The plasmatic concentration of other proteins produced and secreted by the liver, such as AAT, were, however, not shown to be disrupted in CF patients (Birrer et al., 1994). Hepatic retention is thus unlikely to be the mechanism explaining the normal plasmatic CBG concentrations observed in the CF patients. CBG is therefore possibly released but is then partially cleaved in the plasma. In CF, exacerbated neutrophilic activity with sustained elastase release could be responsible for this cleavage. It is well known that this protease targets CBG, which leads to the release of GC and the irreversible inactivation of CBG (Pemberton et al., 1988). In physiological conditions, neutrophil elastase is inhibited by anti-proteases, mainly AAT. These inhibitors can, however, be briefly overwhelmed by bursts of elastase, which are frequent in CF patients who have several pulmonary exacerbations per year (Stenbit and Flume, 2011). Interestingly, we showed an increase of elastase/AAT complex in our CF plasmas (**Figure 2B**), confirming previous work and supporting the hypothesis that the absence of an increase of plasmatic CBG is explained by an increase in the cleavage of CBG (Meyer et al., 1991). This result is in contrast with the work of Nenke et al. (2016) who showed a decrease of CBG cleavage in AAT deficient patients. The authors, however, postulated that others antiproteases than AAT could inhibit elastase in plasma. Considering that, the bursts of elastase in CF patients are significantly high enough to damage the pulmonary epithelium, elastase does not seem to be counteracted in CF context as it could be in AAT deficiency. The elastase secreted by *P. aeruginosa* cleaves CBG, as recently shown (Simard et al., 2014). Given that CF patients are colonized early, and it is predominantly by this bacteria (Vaincre la Mucoviscidose and Ined, 2017), this protease could also explain the absence of the increase in plasmatic CBG observed.

In CF, the main site of inflammation is the lung: thus, we decided to study the expression of the pulmonary form of CBG. We showed the similarity of the two CBG forms, and the lung and the hepatic CBG had a 100% identity (GenBank: MG652288), confirming the preliminary results from Hammond et al. (1987). No data, however, exists on its expression along the airways. We showed, for the first time, that the pulmonary CBG is expressed in the bronchi and bronchioles but not in the alveoli (**Figure 3**). The role of extrahepatic CBG is discussed in the literature (Sivukhina and Jirikowski, 2014; Meyer et al., 2016). A local and intracellular production of CBG is suggested to work as an inhibitor of GC intracellular actions. Indeed, when GC enter the airway cells, pulmonary CBG would bind these GC, resulting in their containment and the blockade of their anti-inflammatory effects. The hypothesis of an opposite role of hepatic and pulmonary CBG is supported by the fact that, in CF, they are regulated in an opposite manner. The pulmonary CBG level is decreased (**Figure 4**) while the hepatic CBG level is increased (**Figure 1**). This opposite pattern of regulation is also observed in a model of acute pancreatitis in mice (Gulfo et al., 2016). Following the hypothesis on the role of the pulmonary CBG, the decrease observed in the lung of CF patients would mean a decrease in GC blockade. This suggests that the optimization of GC occurs locally, in the CF airway cells.

Interestingly, some studies show that the main GC prescribed for CF patients (prednisone and prednisolone) have very poor to no affinity for CBG (Dunn et al., 1981; Pugeat et al., 1981). This lack of affinity leads to the use of high doses to obtain enough GC at the inflammatory site. GC are, however, not limited exclusively to this site, as if they were binding to CBG, but they act within the whole body with metabolic, immune, and developmental side effects (Oray et al., 2016). Using a GC with a high affinity for CBG could be a way of reducing the side effects, as already suggested in the literature (Chan et al., 2014; Henley et al., 2016). We showed that the concentration of CBG in the plasma of the CF patients was the same as the control subjects and that there was a decrease in pulmonary CBG in CF patients. It seems, therefore, that the binding properties of CBG could be a potential tool to optimize GC treatment in CF patients.

## Author Contributions

AT and PLR performed and designed the experiments, interpreted the results, and wrote the manuscript. JT, NR, and CR performed the experiments. DD provided all of the plasmas and liver samples and wrote the manuscript. SB-L provided all the bronchial, bronchiolar, and alveolar samples and wrote the manuscript. M-PM designed the experiments and wrote the manuscript. AF helped to perform the CBG binding experiments. LG, HC, and OT interpreted the experiments and wrote the manuscript.

## Conflict of Interest Statement

The authors declare that the research was conducted in the absence of any commercial or financial relationships that could be construed as a potential conflict of interest.

## Footnotes

1 www.legifrance.gouv.fr

2 http://graphpad.com/quickcalcs/Grubbs1.cfm
